# Denidation

**Published:** 1875-09

**Authors:** 


Denidation.—Perhaps the most satisfactory and important work connected with our branch of medicine which has been done within the last few years, is that which has reference to the anatomy and physiology of the uterine mucous membrane. But notwithstanding all this excellent labor, the periodic changes which take place in the lining of the uterus during its unimpregnated life still remain undecided. Dr. Tyler Smith, who was one of the earliest to write upon this subject, arrived at very definite conclusions. He had examined the uteri of several women who had died during the catamenial flow, and in each found the mucous membrane of the body of the uterus either in a state of dissolution or entirely wanting. In one case, in which he was assisted by Dr. Handfield Jones, in examining the uterus with a microscope, no traces of the epithelium or of the utricular glands could be found. This evidence has been corroborated and supplemented very recently by the elaborate researches of Dr. John Williams, in a paper which appeared in this Journal, and further evidence confirming his views has come to us from Dr. Barnsfather, of Cincinnati. He says that for a number of years he has been examining microscopically, from month to month, the menstrual discharges of women. From his investigations he finds in all of them exfoliation of the mucous membrane, even from females perfectly healthy. In those suffering from dysmenorrhœa the membrane was hypertrophied, and came away in larger pieces. Dr. Aveling also agrees with these authorities, and has proposed to call this removal of the developed mucous membrane " denidation." Unfortunately, in spite of all this concurrent evidence, complete unanimity does not yet exist, for within the last few months Dr. Engelmann has stated, as the result of his observations, that in not one of the many uteri he has examined after the cessation of the catamenia, has the mucous membrane, or even its superficial layer, been wanting. He believes the tumefied mucous membrane to be removed by a gradual disintegration of its elements and rapid absorption, but admits that in some cases the entire upper stratum may be detached and expelled *in toto* as decidua menstrualis. He does not, however, explain why, when the normal physiological process is increased to morbid intensity, the disintegrating and absorbing processes should fail in removing the developed membrane in what he believes to be the normal manner. There are at present, therefore, two contending theories—the desquamative and the involutive. None deny the existence of periodic changes in the lining of the body of the uterus, preparing it for the reception and retention of the ovum should it be impregnated—a process, which since the time of Harvey has been likened to the nesting of birds, or nidation—the point which remains to be positively decided by the repeated examinations of different observers is the exact way in which the abortive physiological process is terminated. We commend the definite solution of this problem to our readers; at present the preponderance of evidence is with those who believe denidation to be an act of desquamation. If, however, these observers have been so unfortunate as to have always investigated uteri in an abnormal condition, and the involution theory be right, time will undoubtedly prove the fact, and determine the ultimate acceptance of the truth.—*Obstetrical Journal.*

